# Plasma Concentrations of Hepcidin in Anemic Zimbabwean Infants

**DOI:** 10.1371/journal.pone.0135227

**Published:** 2015-08-07

**Authors:** Tatenda G. Mupfudze, Rebecca J. Stoltzfus, Sandra Rukobo, Lawrence H. Moulton, Jean H. Humphrey, Andrew J. Prendergast, SHINE Trial Team

**Affiliations:** 1 Department of International Health, Johns Hopkins Bloomberg School of Public Health, Baltimore, MD, United States of America; 2 Zvitambo Institute for Maternal and Child Health Research, Harare, Zimbabwe; 3 Division of Nutritional Sciences, Cornell University, Ithaca, NY, United States of America; 4 Blizard Institute, Queen Mary University of London, London, United Kingdom; The Pennsylvania State University Hershey Medical Center, UNITED STATES

## Abstract

**Objective:**

Anemia in infancy is a global public health problem. We evaluated the relative contributions of iron deficiency and inflammation to infant anemia.

**Methods:**

We measured plasma hepcidin, ferritin, soluble transferrin receptor (sTfR), alpha-1-acid glycoprotein and C-reactive protein (CRP) by ELISA on archived plasma from 289 HIV-unexposed anemic or non-anemic Zimbabwean infants at ages 3mo, 6mo and 12mo. Among anemic infants, we determined the proportion with iron-deficiency anemia (IDA) and anemia of inflammation (AI). We undertook regression analyses of plasma hepcidin and anemia status, adjusting for sex, age and birthweight.

**Results:**

Anemic infants at 3mo were more stunted and had higher CRP (median 0.45 vs 0.21mg/L; P = 0.037) and hepcidin (median 14.7 vs 9.7ng/mL; P = 0.022) than non-anemic infants, but similar levels of ferritin and sTfR; 11% infants had IDA and 15% had AI. Anemic infants at 6mo had higher hepcidin (median 7.9 vs 4.5ng/mL; P = 0.016) and CRP (median 2.33 vs 0.32mg/L; P<0.001), but lower ferritin (median 13.2 vs 25.1μg/L; P<0.001) than non-anemic infants; 56% infants had IDA and 12% had AI. Anemic infants at 12mo had lower ferritin (median 3.2 vs 22.2μg/L; P<0.001) and hepcidin (median 0.9 vs 1.9ng/mL; P = 0.019), but similar CRP levels; 48% infants had IDA and 8% had AI. Comparing anemic with non-anemic infants, plasma hepcidin was 568% higher, 405% higher and 64% lower at 3mo, 6mo and 12mo, respectively, after adjusting for sex and birthweight (all p<0.01). Plasma hepcidin declined significantly with age among anemic but not non-anemic infants. Girls had 61% higher hepcidin than boys, after adjusting for age, anemia and birthweight (p<0.001).

**Conclusion:**

Anemia is driven partly by inflammation early in infancy, and by iron deficiency later in infancy, with plasma hepcidin concentrations reflecting the relative contribution of each. However, there is need to better characterize the drivers of hepcidin during infancy in developing countries.

## Introduction

Anemia is a serious public health problem affecting 2 billion people worldwide, with infants in developing countries at particularly high risk [1–3]. Iron deficiency plays an important role in the etiology of anemia in infancy, with detrimental long-term effects on cognitive and behavioral development [4–6]. However, infants living in developing countries are also at high risk of recurrent infections and subclinical inflammation in early life [7]. In contrast to iron deficiency, the contribution of inflammation to anemia in infancy has not been well characterized and might explain the limited efficacy of iron supplementation programs in developing countries [8, 9].

Hepcidin, the major hormone regulating iron metabolism, controls iron absorption and distribution by blocking iron efflux from duodenal enterocytes, hepatocytes, the placenta and macrophages [10–12]. Hepcidin production is suppressed during iron deficiency anemia (IDA) to facilitate iron absorption, and is stimulated during inflammation and infection as an innate defense mechanism against iron-dependent extracellular pathogens [12–16]. Because opposing signals from iron deficiency and inflammation regulate hepcidin synthesis, net concentrations of hepcidin reflect the aggregate contribution of each to anemia in infants. Low plasma hepcidin levels (predominantly influenced by iron deficiency) indicate both the body’s need and capacity to absorb iron. High plasma hepcidin levels, in contrast, may indicate anemia of inflammation and explain the limited or potentially harmful effects of iron supplementation often observed during infections [17]. Recent studies [17, 18] suggest that hepcidin levels are the strongest predictor of erythrocyte iron incorporation in African children at increased risk of iron deficiency and inflammation/infection. The added value of hepcidin might therefore be its potential to group anemic infants into iron and non-iron responsive subtypes [17, 18], which has wide public health implications for both the diagnosis and treatment of anemia in low-income settings.

We set out to determine plasma hepcidin levels in a cohort of anemic and non-anemic infants in Zimbabwe. We hypothesized that hepcidin would be driven more by chronic inflammation than by iron deficiency in this setting [19], and that plasma hepcidin levels would therefore be elevated in anemic compared to non-anemic infants over the first year of life. Better understanding the relative contributions of inflammation and iron deficiency to anemia in infancy would provide evidence to help inform iron supplementation programs in developing countries.

## Materials and Methods

This study used data and stored samples from ZVITAMBO, a randomized controlled trial of maternal and neonatal vitamin A supplementation [20]. The ZVITAMBO protocol and primary outcomes have been reported previously [20–23]. Briefly, 14110 mother-infant pairs were recruited within 96 hours of delivery at maternity clinics in Harare, Zimbabwe, between November 1997 and January 2000. Mother-infant pairs were eligible if neither had an acute life-threatening condition and the infant was a singleton with birthweight >1500 g. Maternal HIV status was determined at recruitment, at 6 weeks, 3 months, and then 3-monthly for 12 to 24 months to detect seroconversion [24].

Follow-up was conducted at 6 weeks, 3 months, and then 3-monthly to 12–24 months of age. At each visit, infant weight and height were measured using an electronic scale (Seca Model 727, Hanover, MD, USA), and length board (ShorrBoard, Olney, MD, USA), respectively. Weight-for-age (WAZ) and height-for-age (HAZ) Z-scores were calculated using WHO Anthro version 3.0.1. Hemoglobin was measured in real time using the HemoCue hemoglobinometer (HemoCue, Mission Viejo, CA) in a random subsample of infants, for a total of 535 infants born to HIV-negative women [25]. Caregivers of infants with hemoglobin < 70 g/L were encouraged to take the child to a health facility for assessment.

### Study subjects for hepcidin study

We conducted a cross-sectional study of anemic and non-anemic infants at 3, 6 and 12 months of age. Non-anemic infants comprised healthy, HIV-unexposed infants at each age, to generate normative hepcidin values for African infants. These healthy infants were selected based on gestational age >37 weeks, birth weight > 2500 g, no abnormal iron indicators (defined as hemoglobin < 105 g/L at 3 and 6 months and < 100 g/L at 12 months, serum ferritin < 12 μg/L, sTfR > 8.3 mg/L), no evidence of inflammation (defined as AGP > 1 g/L or CRP > 5 mg/L) and no acute illness (defined as diarrhea or fever in the prior week or measles in the prior 3 months). These normative data have previously been published and are included in this analysis as reference values for comparison with anemic infants [26]. Anemic infants at each age were selected based on gestational age >37 weeks, birth weight > 2500 g and hemoglobin (defined as <105 g/L at 3 and 6 months, and <100 g/L at 12 months of age, in line with previous studies [25, 27, 28]), and availability of cryopreserved plasma (>120 uL). The criteria used to diagnose anemia were modified from those of the World Health Organization [29] which are extrapolated from older children, because of the growing literature supporting lower hemoglobin cut-offs in infancy [25, 27, 28].

**Fig 1:**
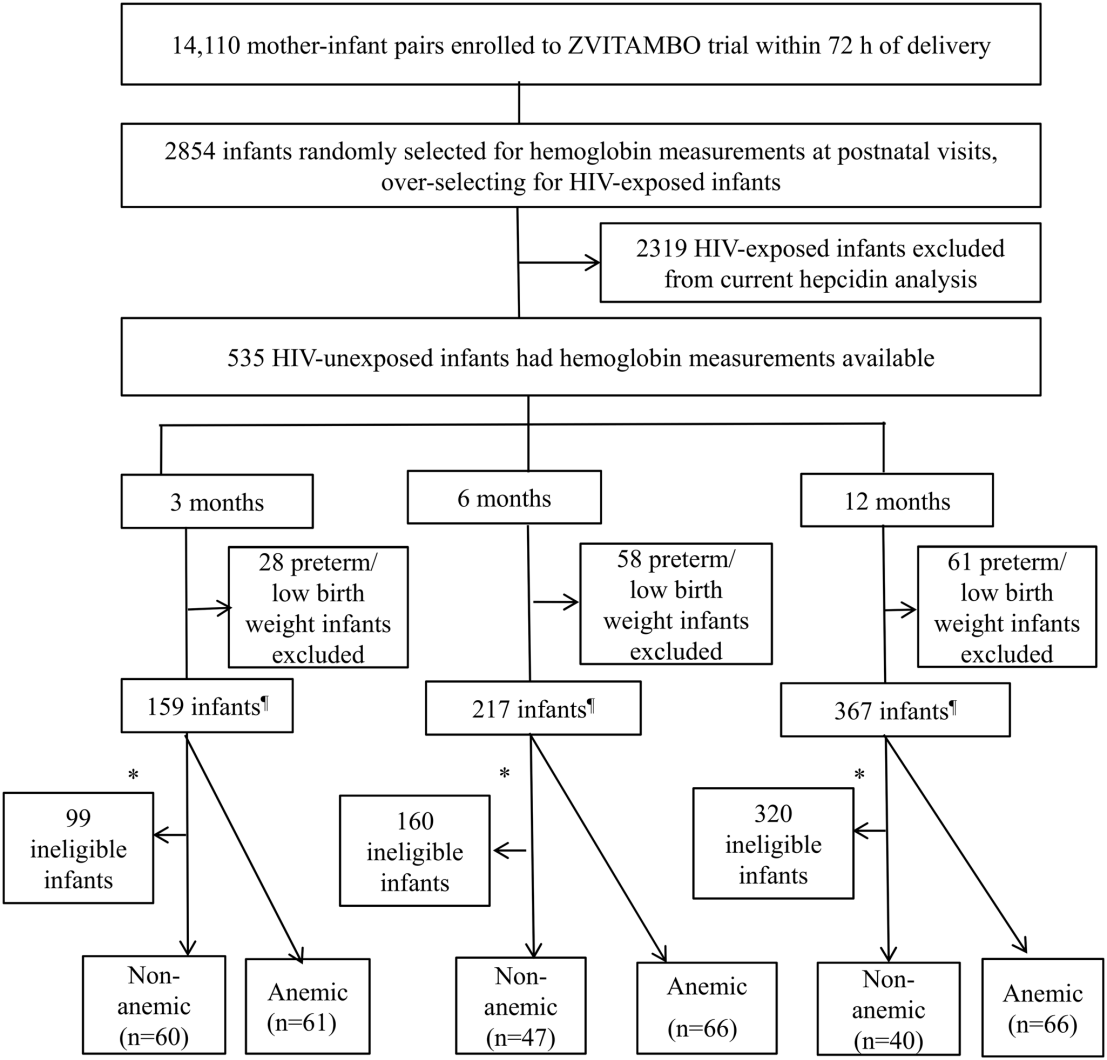
Selection of infants into the hepcidin substudy. ^¶^ HIV-unexposed infants with gestational age >37 weeks, birth weight > 2500 g and available plasma samples (>120 μL). *Non anemic infants were selected based on no abnormal iron indicators (defined as hemoglobin < 105 g/L at 3 and 6 months and < 100 g/L at 12 months, serum ferritin < 12 μg/L, sTfR > 8.3 mg/L), no evidence of inflammation (defined as AGP > 1 g/L or CRP > 5 mg/L) and no acute illness (defined as diarrhea or fever in the prior week or measles in the prior 3 months). Anemic infants at each age were randomly selected from 62, 77 and 85 eligible infants at 3, 6 and 12 months based on hemoglobin (defined as <105 g/L at 3 and 6 months, and <100 g/L at 12 months of age.

We therefore selected anemic and non-anemic infants at three cross-sectional ages, resulting in 6 infant groups, as shown in Fig 1. All non-anemic infants meeting the inclusion criteria above were included in the study (n = 60 at 3 mo; n = 47 at 6 mo and n = 40 at 12 mo). Anemic infants with a gestational age >37 weeks and birth weight > 2500 g were randomly selected from among 62, 77 and 85 eligible infants at 3, 6 and 12mo of age, respectively (n = 61 at 3 mo; n = 66 at 6 mo and n = 66 at 12 mo). The cohort comprised 289 unique infants: 243 infants contributed data at one time point, 41 infants contributed data at two time points and 5 infants contributed data at all three time points (4 anemic and one non-anemic infant).

### Laboratory assays

Plasma levels of soluble transferrin receptor (sTFR) and ferritin were measured by enzyme immunoassay (Ramco Laboratories Inc, Houston, TX); plasma alpha-1-acid glycoprotein (AGP) and C-reactive protein (CRP) were measured by ELISA (R&D Systems Inc, Minneapolis, MN).

Hepcidin was measured in plasma by competition ELISA, using the hepcidin-25 (human) enzyme immunoassay kit (S-1337; Bachem, San Carlos, CA) with detection range 0.02–25 ng/mL, according to the manufacturer’s protocol. Plasma was diluted 1 in 4 in peptide-cleared human serum. Standards were run in duplicate and samples in singlicate, according to the manufacturer’s protocol. Samples giving readings outside the linear region of the curve were re-run at alternative dilutions. The intra-assay CV was mean 6.3% (range 5.7–6.9%), and inter-assay CV was 6.3%.

Iron deficiency was defined according to WHO guidelines, using a combination of low ferritin (< 12 μg/L in the absence of inflammation (CRP ≤ 5 mg/L) or < 30 μg/L in the presence of inflammation (CRP > 5 mg/L)) [30, 31] plus evidence of iron depletion in the tissues, as evidenced by a sTfR/log_10_ ferritin (sTfR-F) index >2 [18]. Anemia of inflammation (AI) was defined as hemoglobin < 105 g/L at 3 and 6 months or < 100 g/L at 12 months and CRP >5 mg/L, with no iron deficiency (ferritin > 30 μg/L and sTfR/log_10_ ferritin (sTfR-F) index < 2).

### Statistical analysis

Baseline variables and biomarker concentrations are reported as median with interquartile range (IQR), or mean with standard deviation (SD). Comparisons between groups were made using Mann-Whitney, t test and Chi-squared tests. Ferritin and plasma hepcidin values below the detection limits of 0.59 μg/L and 0.02 ng/mL, respectively, were imputed using the limit of detection (LOD)/√2, thereby assigning a value of 0.42 μg/L for ferritin and 0.014 ng/mL for hepcidin [32]. Hepcidin consensus values (hepcon1) were generated using the algorithm developed by Kroot *et al*, to allow for comparisons between studies [33]. Iron (ferritin and sTFR) and inflammatory biomarkers (AGP and CRP) and plasma hepcidin were log-transformed for regression and correlation analyses. Multivariate regression analysis was used to test the hypothesis that plasma hepcidin levels would be elevated in anemic compared to non-anemic infants over the first year of life, adjusting for plausible confounders (age, sex and weight-for age-z score (WAZ) at birth). Generalized estimation equations (GEE) were used to adjust for within-child correlations among infants who contributed data to more than one time point. All statistical analyses were performed using STATA version 12 (StataCorp, College Station, TX).

### Ethics statement

This study was carried out in accordance with the Declaration of Helsinki. The original ZVITAMBO trial and this sub-study were approved by the Medical Research Council of Zimbabwe and the Committee on Human Research of The Johns Hopkins Bloomberg School of Public Health. Written informed consent was obtained from mothers at recruitment. Data may be made available by contacting the corresponding author.

## Results

### Characteristics of anemic and non-anemic infants

**Fig 2:**
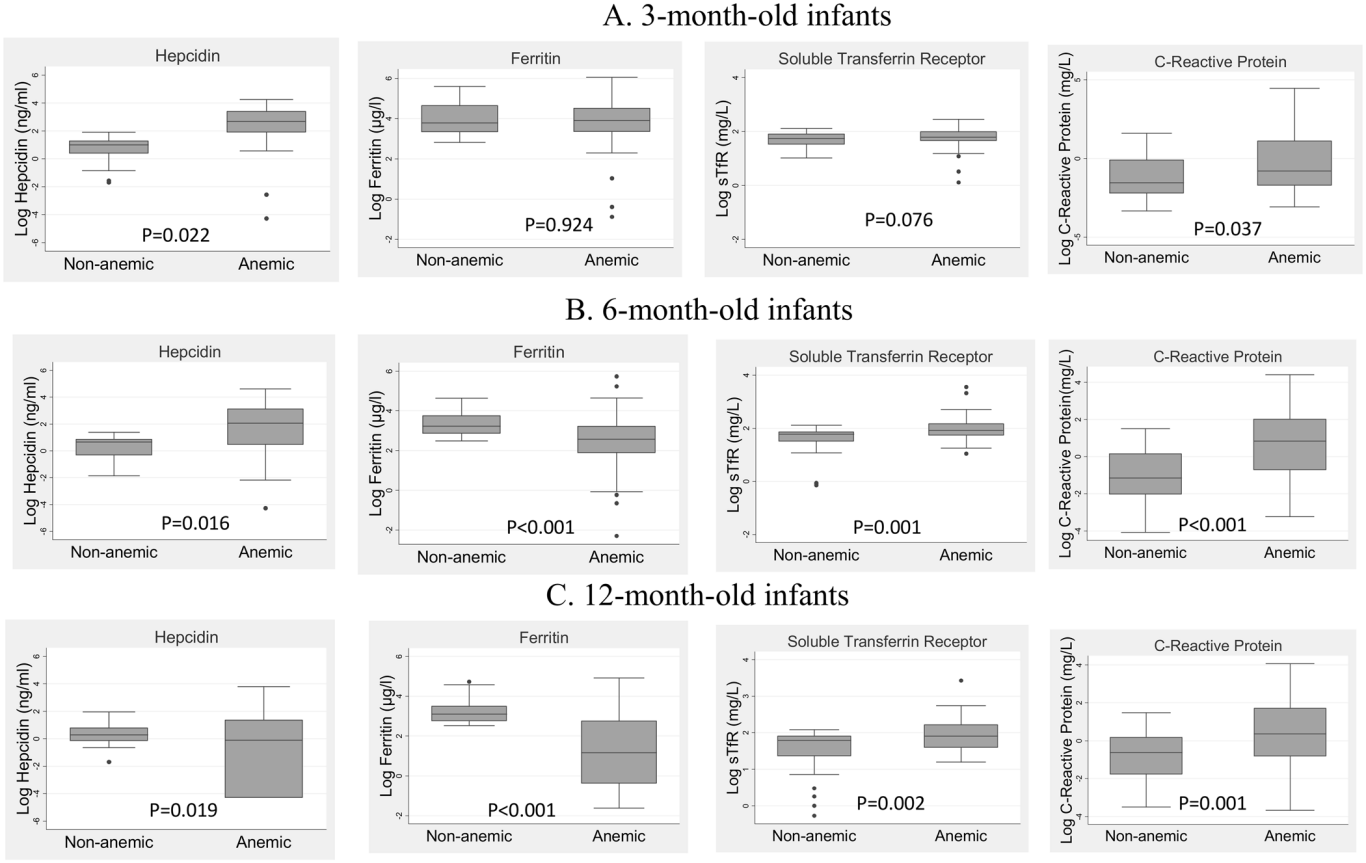
Biomarker concentrations in anemic and non-anemic infants. Graphs show the plasma concentrations of hepcidin, ferritin, soluble transferrin receptor and C-reactive protein (CRP) in infants at (A) 3 months, (B) 6 months and (C) 12 months of age. The boxes indicate the 25^th^ and 75^th^ percentiles and the horizontal line indicates the median. Hepcidin, ferritin and CRP are expressed on a log scale. Full data for each biomarker are also shown in Table 2.

Baseline characteristics of anemic and non-anemic infants at each age are shown in Table 1. At 3 months, infants who were anemic were more stunted (ie lower length-for-age Z score), and had significantly higher plasma concentrations of CRP and hepcidin compared to non-anemic infants (Fig 2A). However, ferritin and sTfR levels were not significantly different between anemic and non-anemic infants at 3 months. Overall, 11% infants at 3 months had iron deficiency anemia and 15% had anemia of inflammation (Table 2).

**Table 1:** Baseline characteristics of non-anemic and anemic infants.

Characteristic	3-month-olds		6-month-olds		12-month-olds	
	Non-anemic N = 60	Anemic N = 61	Non-anemic N = 47	Anemic N = 66	Non-anemic N = 40	Anemic N = 66
***Maternal factors***						
Age, years^1^	27 (6)	24 (6) *	24 (5)	25 (6)	26 (6)	24 (6)
BMI^2^	23.6 (20.8, 25.8)	25.1 (21.5, 27.2)	23.4 (20.5, 25.6)	23.2 (20.2, 26.2)	23.4 (21.0, 26.1)	22.6 (20.5, 26.6)
MUAC, cm^1^	26.4 (2.8)	25.8 (2.7)	25.9 (2.4)	26.0 (3.3)	26.6 (2.7)	25.7 (2.7)
Education, years^2^	11 (9, 11)	11 (9,11)	11 (9, 11)	10 (9,11)*	11 (9, 11)	11 (9, 11)
Household income per month, US dollars^2^	1220 (801, 1694)	913 (730, 1455)	1220 (855, 1830)	913 (610, 1322) *	1221 (879, 2103)	913 (676, 1322)
Hemoglobin, g/L^2^	135 (128, 137)	133 (126, 140)	136 (132, 142)	137 (128, 144.5)	141 (130, 142)	133 (125, 142)
Vitamin A treatment^¥^	55 (33)	53 (32)	49 (23)	47 (31)	50.0 (20)	54.6 (36)
***Infant characteristics***						
Male sex^3^	47 (28)	48 (29)	47 (22)	59 (39)	53 (21)	62 (41)
WAZ at birth^1^	-0.42 (0.79)	-0.50 (0.66)	-0.24 (0.75)	-0.53 (0.80)	-0.18 (0.95)	-0.69 (0.78) *
WAZ at blood sampling^1^	-0.07 (0.91)	-0.26 (0.88)	-0.07 (1.09)	-0.20 (1.17)	-0.39 (1.23)	-0.58 (0.92)
LAZ at birth^1^	0.13 (0.90)	-0.14 (1.03)	0.11 (0.71)	-0.29 (1.09)	0.32 (1.44)	-0.19 (0.96) *
LAZ at blood sampling^1^	-0.32 (0.83)	-0.95 (1.04) *	-0.36 (1.23)	-0.79 (1.08)	-0.84 (1.36)	-1.22 (0.96)
Breastfeeding pattern at 3 months^1^						
Exclusive	26.7 (16)	11.5 (7)	17.0 (8)	10.6 (7)	5.0 (2)	13.6 (9)
Predominant	20.0 (12)	29.5 (18)	14.9 (7)	19.7 (13)	25.0 (0)	18.2 (12)
Mixed	40.0 (24)	41.0 (25)	36.2 (17)	51.5 (34)	42.5 (17)	48.5 (32)
Neonatal vitamin A treatment^¥^ ^1^	52 (31)	44 (27)	55 (26)	47 (31)	52.5 (21)	51.5 (34)

WAZ: weight-for-age Z-score, LAZ: length-for-age Z-score, MUAC: Mid-upper arm circumference, SD: standard deviation, IQR: interquartile range

^1^Values are mean (SD)

^2^Values are median (IQR)

^3^Values are % (n)

^¥^In the ZVITAMBO trial, mother-infant pairs were randomized within 96 h of birth to one of 4 treatment groups (Aa, Ap, Pa, Pp), where ‘A’ was maternal vitamin A supplementation (400,000 IU), ‘P’ was maternal placebo, ‘a’ was infant vitamin A supplementation (50,000 IU) and ‘p’ was infant placebo. Full details of the trial have been published elsewhere [20].

*Denotes p<0.05 for the within age-group pair-wise comparison of anemic and non-anemic infants, using the Mann-Whitney or t test.

**Table 2:** Biomarkers in anemic and non-anemic infants.

tCharacteristic	3-month-olds		6-month-olds		12-month-olds	
	Non-anemic N = 60	Anemic N = 61	Non-anemic N = 47	Anemic N = 66	Non-anemic N = 40	Anemic N = 66
Hemoglobin, g/L^1^	118 (13)	93 (14) *	118 (9)	97 (13) *	117 (11)	89 (11) *
Hepcidin, ng/mL^2^	9.7 (2.5, 19.3)	14.7 (6.8, 29.5) *	4.5 (0.5, 7.3)	7.9 (1.6, 22.7) *	1.9 (0.7, 6.2)	0.9 (0.0, 3.9) *
Hepcidin consensus values ^ψ^ ^2^	6.2 (0.6, 13.7)	10.1 (3.9, 21.7) *	2.2 (-1.0, 4.4)	4.8 (-0.1, 16.3) *	0.1 (-0.8, 3.5)	-0.7 (-1.4, 1.7) *
Ferritin, μg/L^2^	44.1 (27.9, 103.6)	49.3 (28.7, 91.8)	25.1 (17.6, 42.7)	13.2 (6.7, 25.1) *	22.2 (15.9, 32.9)	3.2 (0.6, 15.6) *
Soluble transferrin receptor, mg/L^2^	5.7 (4.6, 6.7)	6.0 (5.3, 7.3)	5.9 (4.6, 6.4)	6.8 (5.7, 8.7) *	6.0 (3.9, 6.7)	6.7 (4.9, 9.2) *
CRP, mg/L^2^	0.21 (0.11, 0.91)	0.45 (0.18, 3.05) *	0.32 (0.13, 1.17)	2.33 (0.49, 7.52) *	0.54 (0.18, 1.20)	1.43 (0.44, 5.52) *
AGP, g/L^2^	0.35 (0.27,0.48)	0.32 (0.23, 0.53)	0.50 (0.30, 0.61)	0.40 (0.27, 0.57)	0.49 (0.38, 0.63)	0.57 (0.42, 0.79)
IDA, % (n)	0 (0)	11 (7)	0 (0)	56 (37)	0 (0)	48 (32)
AI; % (n)	0 (0)	15 (9)	0 (0)	12 (8)	0 (0)	8 (5)

CRP: C-reactive protein. AGP: Alpha-1 acid glycoprotein. IDA: Iron-deficiency anemia. AI: Anemia of inflammation. For all variables, pair-wise comparisons were made using the Mann-Whitney or t test. Anemic infants (hemoglobin <105 g/L at 3 and 6 months, or <100 g/L at 12 month) were categorized as having iron deficiency anemia or anemia of inflammation based on the following definitions: Iron deficiency anemia (IDA) was defined by ferritin < 12 μg/L in the absence of inflammation (CRP ≤ 5 mg/L) or ferritin < 30 μg/L in the presence of inflammation (CRP > 5 mg/L) and sTfR/log10 ferritin (sTfR-F) index >2. Anemia of inflammation (AI) was defined by CRP >5 mg/L with no iron deficiency (ferritin > 30 μg/L and sTfR/log10 ferritin (sTfR-F) index < 2).

^1^Values are mean (SD)

^2^Values are median (IQR)

^ψ^ Hepcidin consensus values were calculated using the algorithm Y = -1.36 + 0.78×hepcidin [33]

* Denotes a raw p<0.05 for the within age-group comparison.

Infants who were anemic at 6 months had lower birth weight and less household income per month compared to non-anemic infants (Table 1). Plasma hepcidin and CRP concentrations were both higher, but ferritin concentrations lower, in anemic compared to non-anemic infants (Fig 2B). There was therefore evidence of both iron deficiency and inflammation in infants at 6 months. Overall, 56% infants had iron deficiency anemia and 12% had anemia of inflammation at 6 months (Table 2).

Infants who became anemic by 12 months had lower birth weight and length than infants who did not become anemic (Table 1). Plasma ferritin levels were significantly lower among anemic compared to non-anemic infants at 12 months (median 3.2 μg/L compared to 22.2 μg/L; p <0.001); Fig 2C. Plasma ferritin levels were significantly lower at this age compared to earlier time-points among the anemic group, whilst ferritin remained fairly constant between 6 and 12 months in the non-anemic group (Fig 2C). Hepcidin levels were lower in anemic compared to non-anemic infants at 12 months, consistent with lower ferritin levels in this age group (Fig 2C). CRP levels were similar in anemic and non-anemic infants at 12 months. Overall, 48% infants had iron deficiency anemia and 8% had anemia of inflammation at 12 months (Table 2).

### Determinants of hepcidin levels during infancy

At 3 months of age, plasma hepcidin concentrations were 568% higher in anemic compared to non-anemic infants (p<0.001), and at 6 months of age, hepcidin levels were 405% higher in anemic compared to non-anemic infants (p<0.001), after adjusting for sex and WAZ at birth. By contrast, at 12 months of age, plasma hepcidin concentrations were 64% lower in anemic compared to non-anemic infants, after adjusting for sex and WAZ at birth at birth (p = 0.004).

Plasma hepcidin concentrations declined significantly with age among anemic infants: compared to infants at 3 months, hepcidin levels were 61% and 97% lower at 6 months and 12 months of age, respectively (p = 0.002 and p<0.001, 2.d.f.); by contrast, the decline in hepcidin with age among non-anemic infants was not significant. Girls had 61% higher plasma hepcidin concentrations than boys (p<0.001), after adjusting for age, anemia status and WAZ at birth. Lastly, a unit increase in WAZ at birth was associated with a 58% increase in plasma hepcidin (p<0.001).

Taken together, we found that age modified the relationship between hepcidin and anemia status. Anemia at the youngest ages (3 and 6 months) was associated with elevated hepcidin, which may in part be driven by inflammation in early infancy; anemia at 12 months of age was associated with depressed hepcidin, consistent with iron deficiency later in infancy. Plasma hepcidin concentrations were progressively lower with age in anemic infants, and girls had higher plasma hepcidin levels compared to boys after adjusting for anemia status and WAZ at birth. Increased WAZ at birth was associated with higher hepcidin levels in infancy.

### Associations between hematologic biomarkers

**Fig 3:**
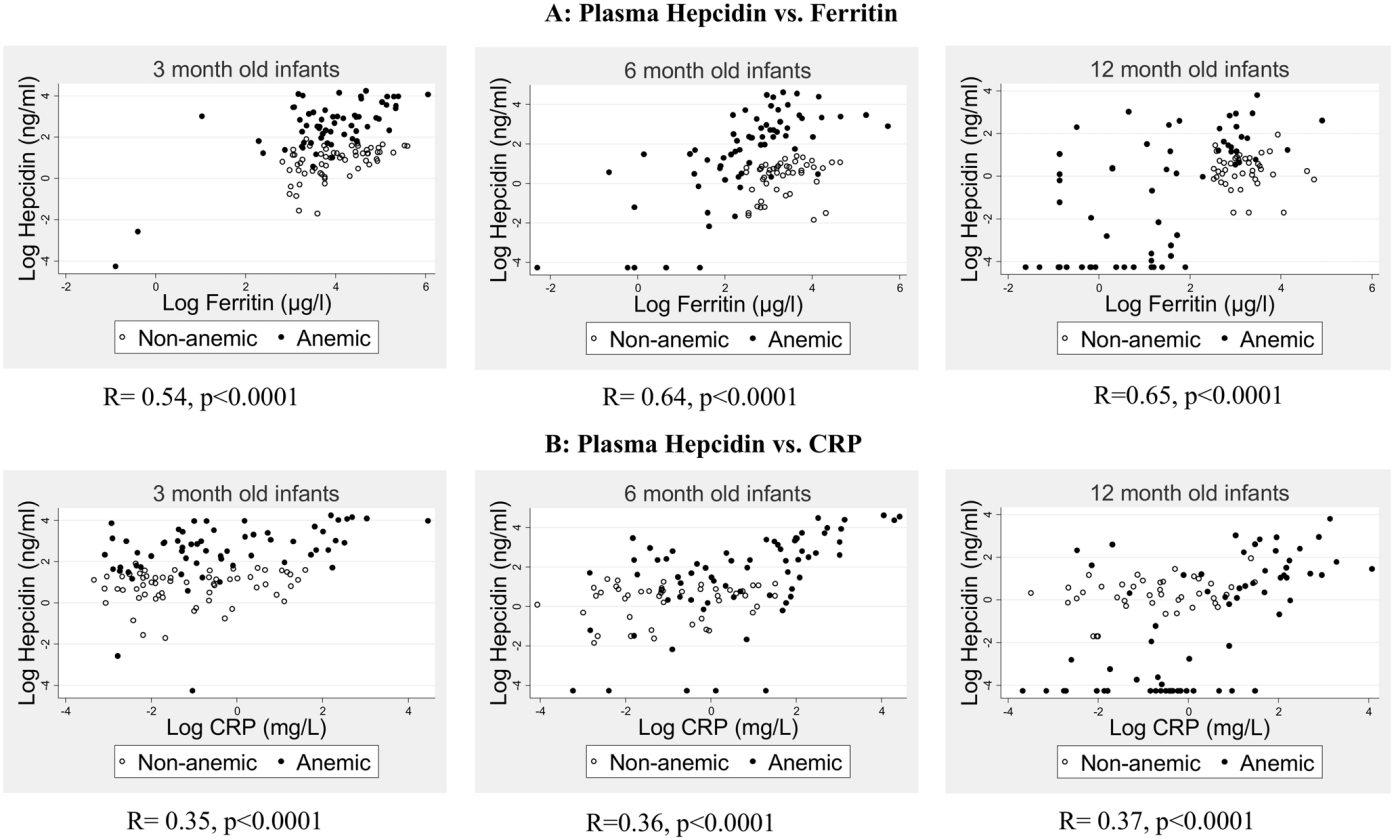
Relationships between hepcidin and other biomarkers. Correlations between (A) plasma hepcidin and ferritin and (B) plasma hepcidin and CRP. Values for non-anemic (empty circles) and anemic (solid circles) infants are combined (p<0.001 for all Spearman correlations). Plasma hepcidin, ferritin and CRP are expressed on a log scale.

Finally, we assessed the relationships between hepcidin, ferritin and CRP during infancy (Fig 3). Anemic and non-anemic groups were combined across ages in order to extend the ranges of biomarker concentrations. Plasma hepcidin levels were positively correlated with both ferritin and CRP (p<0.001 for all Spearman correlations), with the association stronger for ferritin (R = 0.54, 0.64 and 0.65 at 3, 6 and 12 mo, respectively) than for CRP (R = 0.35, 0.36, and 0.37 at 3, 6 and 12 mo, respectively; Fig 3).

## Discussion

Hepcidin is the master regulator of iron homeostasis and plasma concentrations are therefore likely to reflect both the dynamic iron metabolism typical of infancy and the multifactorial etiology of anemia in developing countries [34–37]. We measured plasma hepcidin concentrations and other hematologic biomarkers in a well-characterized cohort of infants in Zimbabwe, where the prevalence of anemia (74% by 12 months of age) is typical of many sub-Saharan African countries [38]. The main objective of this study was to compare plasma hepcidin levels between anemic and non-anemic infants over the first year of life. We show that the relationship between plasma hepcidin levels and anemia status in infancy is modified by age, with elevated hepcidin evident in the first half of infancy and depressed hepcidin in the second half of infancy. We also show that plasma hepcidin concentrations are progressively lower at older ages in anemic infants, that plasma hepcidin levels are higher in girls than boys, after adjusting for anemia status and age, and that larger birthweight is associated with higher hepcidin in infancy.

We hypothesized that plasma hepcidin levels would be predominantly driven by chronic inflammation and hence elevated in anemic infants compared to non-anemic infants over the first year of life. However, contrary to our hypothesis, the association between plasma hepcidin levels and anemia status changed with age. Anemia was only associated with elevated hepcidin in the first half of infancy; by the end of infancy, anemia was associated with low hepcidin concentrations and appeared to be predominantly driven by iron deficiency.

The drivers of elevated hepcidin among anemic infants in the first 6 months of life are unclear. Infants living in developing countries are at high risk of recurrent infection and subclinical inflammation, which is apparent soon after birth [19]. In contrast, endogenous iron stores are less likely to become depleted in the first half of infancy in full term, normal weight infants [39]. Consistent with this, we found that plasma concentrations of CRP and hepcidin were higher in anemic compared to non-anemic infants at 3 months of age. We previously showed that elevated inflammatory biomarkers as early as 6 weeks of age were associated with poor linear growth in this Zimbabwean cohort [19]. It is noteworthy that infants early anemia in the current study were more stunted at 3 months than non-anemic infants, suggesting that inflammation may in part provide a mechanistic link between these two conditions. We were unable to investigate the cause of chronic inflammation in this cohort; however, recurrent infections, environmental enteric dysfunction and residual inflammation from the intrauterine period may all contribute. However, despite overall higher concentrations of CRP and hepcidin in the anemic group, only 15% infants at 3 months fulfilled the strict criteria for anemia of inflammation. Hepcidin synthesis is governed by multiple factors apart from inflammation, including iron stores, hypoxia and erythropoiesis, and hepcidin release into the peripheral circulation is regulated by other proteins including hemojuvelin, hereditary hemochromatosis protein, transferrin receptor 2, matriptase-2 and neogenin [40–42].

The complex homeostatic network controlling hepcidin concentrations is therefore not fully understood, particularly during the developmentally dynamic period of infancy. Whilst inflammation in early life is one potential driver of elevated hepcidin, it is interesting that inflammatory markers were even higher at the end of infancy, yet hepcidin levels were reduced in anemic compared to non-anemic infants. Since we measured only a limited panel of biomarkers, we are unable to evaluate further the mechanism underlying elevated hepcidin in early in infancy and future studies should investigate this further.

By 6 months of age, CRP concentrations remained elevated in anemic compared to non-anemic infants and 12% infants fulfilled criteria for anemia of inflammation; however, plasma hepcidin levels were more modestly elevated in anemic infants at 6 months, compared to infants at 3 months. Plasma hepcidin levels in this age group may partly reflect the capacity for hepcidin to integrate opposing signals from iron deficiency and inflammation, because over half of infants at 6 months of age had iron deficiency anemia. Hepcidin values in this age group therefore appear to represent a crossover period from inflammation and other drivers in early infancy to classical iron deficiency in later infancy.

Infants who were anemic at 12 months were more stunted at birth and had lower birth weight compared to non-anemic infants. While plasma ferritin levels remained fairly constant beyond 6 months in non-anemic infants, ferritin continued to drop in anemic infants, to reach a nadir of median 3.2 μg/L by 12 months. Almost half of anemic infants were iron deficient at 12 months and plasma hepcidin levels were lower in anemic compared to non-anemic 12-month-old infants, consistent with classical iron deficiency anemia. Although we observed modest increases in AGP and CRP with age, contrary to our hypothesis, plasma hepcidin levels by the end of infancy (12 months) appeared to be predominantly driven by low plasma ferritin levels.

In a similar study of older African refugees (mean age 8.0 years), urinary hepcidin levels were significantly lower in children with iron deficiency anemia compared to those without IDA [43]. Our results support the feedback mechanism between iron deficiency anemia, and/or low plasma ferritin, and suppression of hepcidin production [34, 43]. Lower plasma hepcidin levels reflect both increased dietary requirement for iron in the second half of infancy, and an increased ability to efficiently absorb iron in older infants [44]. Infants experience major physiologic changes in iron status with age. The depletion of endogenous iron stores and inadequate dietary iron during the second half of infancy is associated with a rising prevalence of iron deficiency anemia with age [39]. Consistent with the rising prevalence of iron deficiency with age in this cohort, plasma hepcidin concentrations were progressively lower at older ages in anemic infants [44]. Our results therefore underscore the predominance of iron deficiency as the leading cause of anemia in older infants.

We also observed lower plasma hepcidin levels in boys compared to girls, after adjusting for anemia status, age and birthweight. We previously hypothesized that lower hepcidin concentrations in boys might be a physiologic response to inherently lower iron stores [26, 45]. Consistent with this hypothesis, log plasma ferritin concentrations were 66% higher in girls compared to boys in unadjusted models and 30% higher after adjusting for anemia status and age. Lower plasma hepcidin and ferritin levels in boys may explain the increased vulnerability to anemia among boys during infancy [46–49]. Lastly, increased birthweight was associated higher hepcidin levels, consistent with previous research that has shown low birthweight infants to be at higher risk for iron-deficiency anemia among those born with better nutritional status [25, 50]

Although we assayed samples at 3, 6 and 12 months of age, the cross-sectional nature of our study limits our ability to make inferences about the trajectory of hepcidin over the first year of life. Furthermore, we conducted exploratory analyses of the differences between anemic and non-anemic infants; however, the multiple comparisons limit the statistical significance of our results. We had very limited data on the type of anemia in each infant group because we did not assess other red cell indices and did not have information on several potential underlying causes of anemia. However, the prevalence of sickle cell anemia in southern Africa is less than 1% [51] and malaria is non-endemic in greater Harare. Nonetheless, we were unable to explain a proportion of anemia at each age and further studies are needed to better understand the multifactorial nature of this highly prevalent public health problem.

In summary, anemia appears to be driven, at least in part, by inflammation early in infancy, and by iron deficiency later infancy, with plasma hepcidin concentrations reflecting the relative contribution of each at different ages. Plasma hepcidin levels were progressively lower with age and lower in boys compared to girls, consistent with reduced iron stores. Future studies should further explore the drivers of hepcidin during infancy, the role of plasma hepcidin in the pathogenesis of anemia and the utility of hepcidin as a diagnostic tool for determining the etiology of anemia and likely response to iron supplementation in developing countries.
